# A Gentleman with Anemia and Cholestasis

**DOI:** 10.1155/2010/536207

**Published:** 2010-12-08

**Authors:** Siu-Tong Law, Wai-Ki Lee, Michael Kin-Kong Li, Ka-Ho Lok

**Affiliations:** ^1^Division of Gastroenterology and Hepatology, Department of Medicine and Geriatrics, Tuen Mun Hospital, Tuen Mun, Hong Kong; ^2^Department of Pathology, Tuen Mun Hospital, Hong Kong

## Abstract

Primary sclerosing cholangitis is a rare cause of cholestasis caused by progressive inflammation and fibrosis of both intrahepatic and extrahepatic bile ducts leading to multifocal ductal strictures. Herein, we report a case of primary sclerosing cholangitis and inflammatory bowel disease. The concomitant diagnosis of these two diseases is not typical. The management includes the treatment of inflammatory bowel disease and potential complications of primary sclerosing cholangitis, including dominant strictures of bile duct, portal hypertension, gallbladder diseases, cholangiocarcinoma, and colonoscopic surveillance.

## 1. Introduction

Primary sclerosing cholangitis is a rare cause of chronic cholestasis in adult with prevalence of about 1–5/100,000 in Caucasian inhabitants. There is a close association with inflammatory bowel disease. The clinical course is, yet variable and unpredictable, slowly progressive and develops into biliary cirrhosis and corresponding complications. Therapeutic measures aim in improving bile flow to prevent the progression of biliary obstruction and liver transplantation is the treatment of choice in advanced stage of the disease.

## 2. Case Report

A 31-year-old man was admitted to the hospital because of hypochromic microcytic anemia 

He had chronic nonspecific epigastric pain for the past six months which had bloating sensation without radiation and any relationship to meal. He consulted a private practitioner. The complete blood picture showed that the hemoglobin was only 6 g/dl, and so he was referred to our unit for further management. His appetite reduced with subjective weight loss in the past three months'time. His bowel opening increased up to two times per day more loose in nature. All being along there was no per rectal bleeding. His past health was well except for taking herbal medicine for acne for the past seven months. 

On examination he was pale with the absence of stigmata of chronic liver disease. The abdominal examination showed hepatomegaly. Laboratory data were as follows: hemoglobin, 4.3 g/dL (normal: 13.4–17.2); mean cell volume, 49.6 fl (normal: 83–98); white blood cell count, 9/mm^3^ (normal: 3.9–10.7); platelet count, 508/mm^3^(normal: 152–358); total bilirubin, 17 umol/L (normal: 5–20); alkaline phosphatase, 1541 IU/L (normal: 46–127); *γ*-glutamyl transpeptidase, 366 IU/L (normal: 12–57); alanine aminotransferase, 102 IU/L (normal: 10–57); albumin, 34 g/l (normal: 35–50); globulin, 40 g/l (no reference); iron saturation, 1% (normal: 20–55); hemoglobin A2, 4.8% (normal: 1.6–3.5). 

The preliminary investigations revealed that he had severe iron deficiency anemia coexisting with *β* thalassaemia trait and cholestatic liver derangement. The esophagogastroduodenoscopy (OGD) showed no abnormality down to the third part of duodenum. Early colonoscopy performed one week later showed that the colonic mucosa was erythematous with loss of vascular pattern and multiple small superficial ulcerations in which the proximal parts including ascending and transverse colon were more severely affected. The mucosa of terminal ileum, sigmoid, and rectum was endoscopically normal looking. The histology revealed that there was inflammatory cell infiltration at lamina propria of terminal ileum, and colon, the latter also having distorted cryptal architecture. The abdominal ultrasonography showed that the liver was enlarged with 16.7 cm of span length and dilated intrahepatic and common bile duct. Further, relevant blood tests showed that the perinuclear antineutrophil cytoplasmic antibodies (p-ANCAs) were present while other autoimmune antibody (including antinuclear, antimitochondrial, antismooth muscle antibody), HBsAg, anti-HCV and HIV antibody were absent.

**Figure 1:**
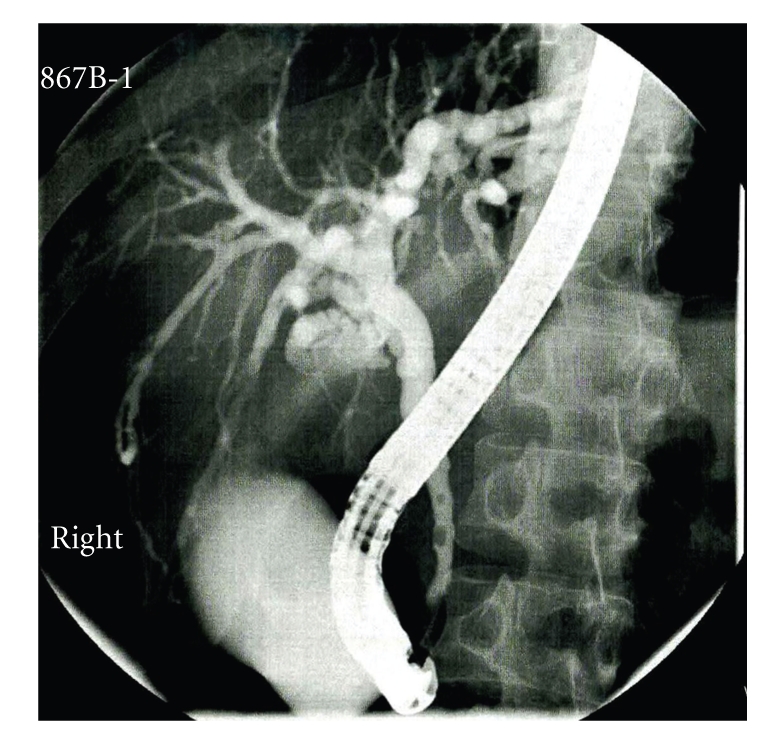
ERCP showing irregular wall contours, variable intrahepatic stenoses, and two distal common bile duct stones.

**Figure 2:**
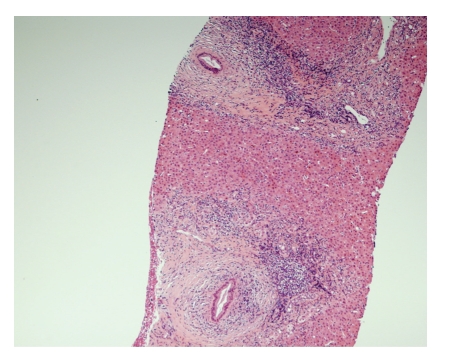
Marked expansion of portal tracts by fibrosis and inflammation (low-power view).

**Figure 3:**
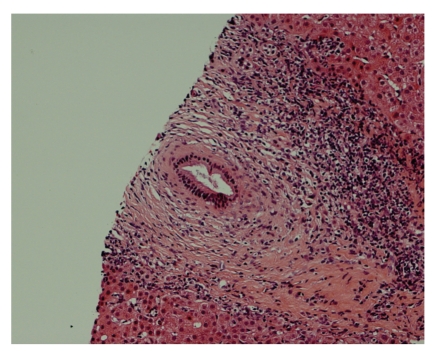
Concentric fibrosis and portal tract inflammation (intermediate-power view).

**Figure 4:**
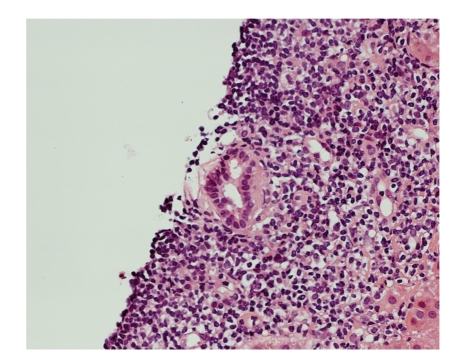
Atrophic interlobular bile duct (high-power view).

During the next 3 days, his hemoglobin was topped up to 8.7 g/dL by two units of packed cell. Then endoscopic retrograde cholangiopancreatography (ERCP) was performed and found multiple irregularities over bilateral intrahepatic bile ducts: common bile duct was not dilated but with two small stones distally (Figure 1). These stones were extracted after papillotomy. The liver biopsy was also performed and revealed that the portal tracts had mixed inflammatory infiltrate, some interlobular bile ducts having concentric, laminated (onion-skin) fibrosis around them, and focal bile ductular proliferation. These were consistent with primary sclerosing cholangitis, Stage III (Ludwig) (Figures 2, 3 and 4).

Therefore, this gentleman was diagnosed to have primary sclerosing cholangitis coexisting with ulcerative colitis. He was put on medications including ursodeoxycholic acid 500 mg bd, enteric coated mesalazine 2000 mg bd, and iron supplement. He was regularly followed up for the past 4 months and his condition was stable in which his hemoglobin remained static with hemoglobulin level around 9 g/dl and the alkaline phosphatase improved to 204 U/L.

## 3. Discussion

The diagnosis of primary sclerosing cholangitis (PSC) in this patient is established by the biochemical profile of chronic cholestasis, typical strictures and pruning of the biliary tree upon cholangiography, and ring fibrosis around the bile ducts in liver biopsy. The coexisting iron deficiency anemia should lead to the suspiciousness of coexisting inflammatory bowel disease which was confirmed by colonoscopy in our case.

Firstly reported by Dr. K. Delbet in 1924 by constellation of symptoms including pruritus and cholestatic liver pattern, PSC is characterized pathologically by the progressive, fibrous-stenosing and obliterating, predominantly segmental inflammation of the intra- and extrahepatic bile ducts and preferably affects male with a maximum age of around 25–45 [1]. About 80% of patients show both intrahepatic and extrahepatic involvement; 20% showed only extrahepatic involvement [2].

It is likely an immune-mediated disease with a wide range of autoantibodies detected in which p-ANCA, having the highest prevalence and detected in our patient, occurs in 80% of patients. Although it does not correlate with the activity of PSC, it may draw attention to colon involvement [3].

For diagnostic imaging, the typical findings of ERCP and MRCP in PSC are pearl-string-like changes of the bile ducts with intermittent, diffusely distributed, multiple, and irregular strictures of different length. Nowadays MRCP replaces the role of ERCP in the diagnosis of PSC because it is noninvasive with high sensitivity and specificity (both are greater than 80%) whereas ERCP can lead to potential serious complications such as pancreatitis and bacterial cholangitis [4]. As MRCP is not easily available in our centre, ERCP was performed instead, and the typical strictures and pruning of the biliary tree were demonstrated and two stones were found concurrently and removed uncomplicatedly in the same session. The liver biopsy of our case revealed the typical features of PSC. In fact, a liver biopsy is not required in the presence of typical cholangiographic features of PSC unless a small duct PSC is suspected because the disease localizes in intrahepatic ducts [5]. It was performed in our case because he was young with quite advanced laboratory parameters and had an elevated serum aminotransferase. Thus an accurate staging of the disease and exclusion of a PSC-autoimmune hepatitis (PSC-AIH) are warranted. A clinical entity called autoimmune pancreatitis (AIP-SC), characterized by a lymphoplasmacytic infiltrate around pancreatic duct and elevated serum IgG4, can cause stricturing in intrahepatic and extrahepatic bile duct similar to that present in PSC. Distinguishing from PSC, both PSC-AIH and AIP-SC are responsive to corticosteroid [6]. 

PSC is strongly associated with inflammatory bowel disease (IBD) in which up to 80% are associated with ulcerative colitis (UC), and only 10% Crohn's disease with the latter is usually diagnosed before PSC [7]. As IBD in PSC may be asymptomatic, and therefore any newly diagnosed PSC should have full colonoscopy with biopsies. Although IBD may be diagnosed at any time during the course of PSC, the concomitant presentation like in our case is not typical [8]. There are several clinical and endoscopic features of IBD in PSC distinctive from ordinary IBD in which the former usually has more pancolitis, rectal sparing, and backwash ileitis. These mentioned features are present in our patient. In addition, IBD has more frequently quiescent and prolonged subclinical course [9, 10]. The medical therapy does not differ from that of IBD without PSC. The indications for urgent surgery are acute severe colitis not responding to medical treatment, toxic dilatation, perforation, or hemorrhage while the indications for elective surgery fall into two groups: failure of medical therapy and dysplastic/malignant change in the colon. Proctocolectomy with ileal pouchanal anastomosis is now the procedure of choice because it has the advantage of both removing the diseased colon and avoiding a permanent ileostomy. Patients with UC and PSC are at higher risk of colorectal neoplasia compared with those with UC alone, with odd ratio 4.79 and predilection for right-side distribution [11]. Thus, surveillance colonoscopy with biopsies at up to two-year intervals is recommended. 

There is no effective medical treatment of PSC; the routine use of ursodeoxycholic acid (UDCA) is not recommended because of unclear benefit and possible serious adverse effects in high dose (28–30 mg/kg/day) [12]. Treatment with corticosteroid and other immunosuppressant agents does not show any beneficial effect. However, our case received UDCA of the dose around 15 mg/kg/day, and his serum liver ductal enzymes seemed improving, but the long-term effect needs to be observed. 

The management of our patient should also include management of potential complications including portal hypertension, dominant strictures of bile ducts, gallbladder diseases, cholangiocarcinoma, and colorectal neoplasia. 

 Concerning portal hypertension, about 40% newly diagnosed cases have esophageal varices, and its management does not differ from non-PSC patients [13]. The dominant stricture in PSC is defined as a stenosis with a diameter of less than 1.5 mm in common bile duct or of 1 mm in intrahepatic ducts [14]. It happens in about 50% of patients during the followup, and the common presentations are jaundice, pruritus, right-upper quadrant pain, and elevated serum bilirubin. The management is to relieve biliary obstruction by endoscopic balloon dilatation with or without stent placement preceded by the brush cytology and biopsy at the stricture site to exclude a superimposed malignancy [15]. In an early case series, the gallbladder abnormalities are frequently observed, including gallstones (26%), PSC involving gallbladder (15%), and neoplasm of gallbladder (4%) [16]. Therefore, an annual ultrasound of biliary system is recommended to detect mass lesions in the gallbladder. In our case, the coexisting common bile duct stones might be formed in situ or migrated from the gallbladder. Lastly, PSC is a risk factor for cholangiocarcinoma, with 10% ten-year cumulative incidence [17]. It is a difficult task to distinguish it from the benign stricture. Because of lack of any diagnostic test, the diagnosis of cholangiocarcinoma relies on the following features: contrast-enhanced mass lesion in imaging, positive biopsy/cytology, or highly elevated CA 19-9 in case of borderline resulting from imaging and histology. For early stage of cholangiocarcinoma, surgical resection is indicated in patient of good liver functional condition while liver transplantation following neoadjuvant therapy is an option in case of poor liver reserve [18]. 

The natural course of PSC depends on the respective stage at the time of diagnosis, and its survival rate is around 60% after 6 years [19]. Because of the absence of effective treatment available, there are several prognostic models to predict the clinical outcome, such as Mayo score, which is shown to be useful in predicting the clinical course particularly the early stage of PSC, and this score includes age, bilirubin, serum aminotransferase, albumin, and history of variceal bleeding [20]. Liver transplant indications for patient with PSC include liver failure and several unique indications such as intractable pruritus, recurrent bacterial cholangitis, and cholangiocarcinoma. The appropriate time for referral for liver transplantation includes one of the following: Child-Pugh score of seven, model for end-stage liver disease (MELD) of 10, or any complication of portal hypertension. Overall, the results of liver transplantation are good with 70% in 10-year survival rates [21, 22]. The risk of developing colonic neoplasia in ulcerative colitis still persists after transplantation, and therefore annual colonoscopic surveillance is still warranted. In our patient, he has been regularly followed up with stable condition, and he is planned to have regular ultrasound imaging of hepatobiliary system and colonoscopic surveillance.
